# Pathogenesis of Medication-Related Osteonecrosis of the Jaw: Odontogenic Infection-Preceding Type and Osteonecrosis-Preceding Type

**DOI:** 10.7759/cureus.60223

**Published:** 2024-05-13

**Authors:** Yuki Sakamoto, Shunsuke Sawada, Yuka Kojima

**Affiliations:** 1 Department of Oral Surgery, Kansai Medical University Medical Center, Moriguchi, JPN; 2 Department of Dentistry and Oral Surgery/Oral Care Center, Kansai Medical University, Hirakata, JPN; 3 Department of Dentistry and Oral Surgery, Kansai Medical University Hospital, Hirakata, JPN; 4 Department of Dentistry and Oral Surgery, Kansai Medical University, Hirakata, JPN

**Keywords:** infection, odontogenic, non-odontogenic, preceding osteonecrosis, mronj

## Abstract

Introduction

Medication-related osteonecrosis of the jaw (MRONJ) develops from odontogenic infection. However, there are also some cases of MRONJ developing from sites with no teeth, no root canal lesions, or no periodontal disease. This study aimed to retrospectively review radiographic images of MRONJ cases and examine the differences in characteristics between MRONJ suspected to be related to dental infection (odontogenic MRONJ) and MRONJ that occurred without dental involvement or of unknown cause (non-odontogenic MRONJ).

Materials and methods

One hundred and forty-five patients were diagnosed with MRONJ at Kansai Medical University Hospital and Kansai Medical University Medical Center. The following variables were investigated: sex, age, primary disease, MRONJ site, body mass index, smoking habit, diabetes, corticosteroids, type of antiresorptive agent, administration period, CT findings (separation of sequestrum, osteolysis, periosteal reaction, and osteosclerosis), trigger, leukocytes, neutrocytes, neutrophil-lymphocyte ratio, serum albumin, and serum creatinine levels.

Results

In the univariate analysis, significant differences between odontogenic and non-odontogenic MRONJs were found in patients whose primary disease was malignancy, receiving denosumab (DMB), and with short administration period of antiresorptive agent, no osteolysis, periosteal reaction, and serum creatinine level. In multivariate analysis, non-odontogenic MRONJ was significantly more common in patients with no osteolysis and with periosteal reaction.

Conclusion

Non-odontogenic MRONJ tends to occur more frequently in patients treated with high-dose DMB, and there were significantly more cases of non-osteolytic MRONJ without radiographic evidence of osteolysis or with periosteal reactions.

## Introduction

Medication-related osteonecrosis of the jaw (MRONJ) is now widely known as refractory osteonecrosis of the jaw in patients treated with bone resorption inhibitors (ARAs), such as bisphosphonate (BP) and denosumab (DMB). MRONJ is caused by systemic factors, such as medication-related risk factors, immune dysfunction, and genetic factors, as well as local factors such as dentoalveolar operations and concomitant oral diseases such as periodontal disease or periapical pathology [1]. Although most studies report tooth extraction as the major inciting event for MRONJ development, most extracted teeth had pre-existing local infections, such as periodontal or periapical disease. Therefore, local infection rather than the extraction itself has recently been reported as a risk factor for MRONJ [2,3]. Antiresorptive agents and inflammation or infection are necessary and sufficient for the development of MRONJ [4]. Therefore, it is recommended that a dental examination be performed before administering ARA. However, MRONJ often develops even if no tooth is determined to be the source of infection or develops from an area where no tooth is present.

Both ARA action and local infection are involved in the development of MRONJ, but which occurs first is questionable. In 2009, Lesclous et al. proposed the following hypothesis (osteonecrosis precedes osteoporosis): initially, ARA causes inflammation in the bone marrow, which progresses to necrosis in a bacteria-free state. If necrosis spreads and reaches the oral mucosa, the mucosa is destroyed, and the mandibular bone is exposed. Subsequently, oral bacteria invade the exposed necrotic bone, and symptoms of infection appear. Initial aseptic osteomyelitis or osteonecrosis can be diagnosed using bone scintigraphy or MRI [5]. In contrast, it is hypothesized that osteonecrosis results from dental infection caused by periodontal disease or root apex lesions, which are refractory actions of ARA (dental infection-precedent theory) [6]. In clinical practice, most patients with MRONJ present with symptoms of infection, and it is not always clear which pathway caused MRONJ. The former theory suggests that MRONJ cannot be prevented by dental examination and extraction of infected teeth before ARA administration, whereas the latter theory suggests that MRONJ can be prevented by dental examination and extraction of infected teeth before ARA administration. However, only a few studies have focused on the pathogenesis of MRONJ. This study aimed to retrospectively review radiographic images of MRONJ cases in our department and examine the differences in characteristics between MRONJ suspected to be related to dental infection (MRONJ preceding dental infection) and MRONJ that occurred without dental involvement or of unknown cause.

## Materials and methods

Study design and patients

This was a retrospective, observational study. The participants were patients who were diagnosed with MRONJ at Kansai Medical University Medical Center and Kansai Medical University Hospital between September 1, 2013, and March 31, 2021. The inclusion criteria were patients for whom panoramic radiography or CT scans were performed, while the exclusion criteria were patients whose MRONJ site could not be determined from their medical records and patients who refused to participate in this study.

Variables

The following variables were examined: sex, age, primary disease, MRONJ site, body mass index (BMI), smoking habit, diabetes, corticosteroids, type of ARA, administration period, CT findings (separation of sequestrum, osteolysis, periosteal reaction, and osteosclerosis), trigger, leukocytes, neutrocytes, neutrophil-lymphocyte ratio (NLR), serum albumin, and serum creatinine levels. Osteosclerosis was divided into three types according to the classification of Suyama et al.: none, uniform (homogeneous osteosclerosis with uniform radiopaque images), and mixed (many small radiolucent areas within the osteosclerotic image) [7]. Triggers were classified into two categories: odontogenic and non-odontogenic. Non-odontogenic origin was defined as no radiographic findings or clinical symptoms associated with teeth and no events or treatments associated with dental infection, including surgical procedures such as tooth extractions within one year prior to MRONJ onset.

Statistical analysis

All statistical analyses were performed using SPSS, Version 26.0 (Japan IBM Co., Ltd., Tokyo, Japan). Differences between odontogenic and non-odontogenic characteristics were analyzed using the one-way ANOVA or Mann-Whitney U test for continuous data and analyzed using Fisher's exact test for categorical variables. Multivariate analysis was performed using logistic regression with the variables that were significant in the univariate analysis as covariates. Statistical significance was defined as a two-tailed p-value <0.05.

Ethics

This study was conducted in accordance with the Declaration of Helsinki and the Ethical Guidelines for Medical and Biological Research Involving Human Subjects of the Ministry of Health, Labor, and Welfare of Japan. The study was approved by the Institutional Review Board (IRB) of Kansai Medical University Medical Center (approval number: 2022048). As this was a retrospective study, patient-identifiable information was removed, and the research plan was published on the homepages of the participating hospitals' websites, along with an opt-out option in accordance with IRB instructions.

## Results

Patient characteristics

The total number of patients was 145, with 102 odontogenic MRONJ and 43 non-odontogenic MRONJ. Patient characteristics are shown in Table 1. The primary disease was osteoporosis in 88 patients and malignancy in 57. Seventy patients used BP, 53 used DMB, and 22 switched to DMB after the initial use of BP. 

**Table 1:** Patient characteristics Categorical data used a paired t-test and continuous volume data used the Spearman rank correlation coefficient MRONJ: medication-related osteonecrosis of the jaw

Variable		Number of patients
Sex	Male	41
	Female	104
Age (years)	Median/25-75% teil	75/69-80
Primary disease	Osteoporosis	88
	Malignancy	57
MRONJ site	Maxilla	57
	Mandible	84
	Both	4
Body mass index	Median/25-75% teil	21.6/18.7-23.8
Smoking	(-)	140
	(+)	5
Diabetes	(-)	95
	(+)	50
Corticosteroid	(-)	101
	(+)	44
Type or antiresorptive agents	Bisphosphonate	70
	Denosumab	53
	Both	22
Administration period (months)	Median/25-75% teil	31/15-60
Separation of sequestrum	(-)	82
	(+)	60
	Unknown	3
Osteolysis	(-)	19
	(+)	123
	Unknown	3
Periosteal reaction	(-)	133
	(+)	10
	Unknown	2
Osteosclerosis	(-)	41
	Uniform type	59
	Mixed type	42
	Unknown	3
Trigger	Odontogenic	54
	After tooth extraction	48
	Non-odontogenic	43
Leukocytes (/μL)	Median/25-75% teil	6200/4700-8285
Neutrocytes (/μL)	Median/25-76% teil	4099/3021-6127
Lymphocytes (/μL)	Median/25-77% teil	1266/993-1900
Neutrophil-lymphocyte ratio	Median/25-78% teil	2.83/1.99-5.03
Albumin (g/dL)	Median/25-79% teil	3.8/3.5-4.1
Creatinine (mg/dL)	Median/25-80% teil	0.76/0.64-1.01

**Figure 1:**
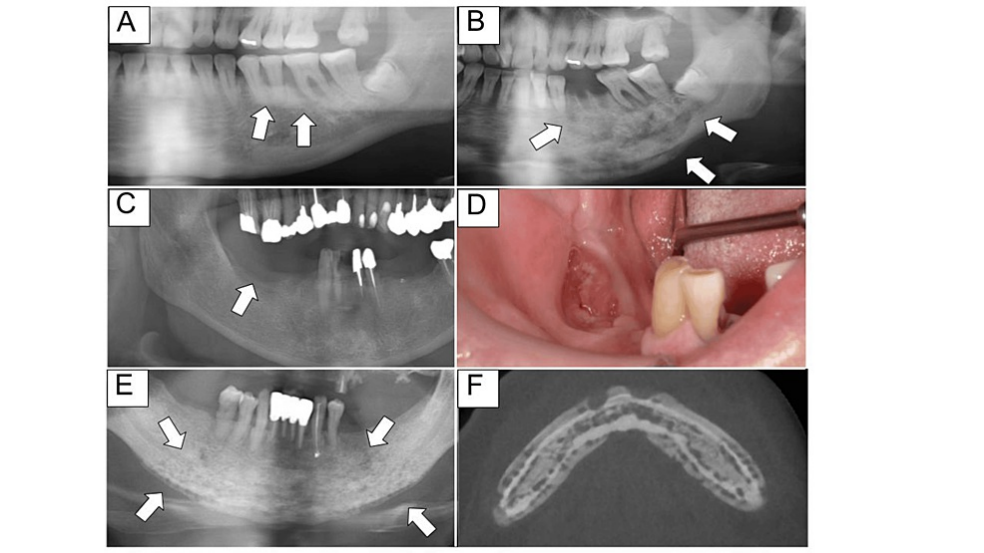
Possible causes of MRONJ development A, B: Severe periodontal disease is observed in the bicuspids and molars, and the development of MRONJ from dental infection is suspected. C, D: MRONJ, possibly caused by an infection from a denture ulcer. E, F: Extensive MRONJ in the body of the bone, remote from the teeth, with no bone exposure to the oral cavity and a cutaneous fistula MRONJ: medication-related osteonecrosis of the jaw

Fifty-four patients developed MRONJ from a dental infection, 48 after tooth extraction, and 43 from sites with no obvious dental infection. Of the 43 patients with non-odontogenic origins, 15 occurred at the denture site, one from mucosal ulceration caused by opposing teeth, two from the palatal torus, and 25 from areas where no teeth existed and the cause was unknown (Figure 1).

Differences between characteristics of odontogenic MRONJ and that of non-odontogenic MRONJ

The characteristics of patients with odontogenic and non-odontogenic MRONJ were compared (Table 2). In the univariate analysis, significant differences were found in patients whose primary disease was malignancy (p=0.027), type of ARA was DMB (p=0.023), duration of treatment was short (p=0.017), no osteolysis (p<0.001), periosteal reaction (p<0.001), and serum creatinine level (p=0.023). In addition, males were slightly more common (p=0.069), and mandibular involvement was slightly more common (p=0.187); however, the difference was not significant.

**Table 2:** Differences of characteristics between odontogenic and non-odontogenic MRONJ: univariate analysis Categorical data used the corresponding t-test; continuous quantitative data used the Spearman rank correlation coefficient. * means significant value MRONJ: medication-related osteonecrosis of the jaw

Variable		Number of patients		P-value
		Odontogenic	Non-odontogenic	
Sex	Male	24	17	0.069
	Female	78	26	
Age (years)	Median/25-75% teil	74/67-81	77/71-80	0.521
Primary disease	Osteoporosis	68	20	*0.027
	Malignancy	34	23	
MRONJ site	Maxilla	43	14	0.187
	Mandible	55	29	
	Both	4	0	
Body mass index	Median/25-75% teil	21.7/17.5-23.8	22.2/19.3-24.0	0.543
Smoking	(-)	98	42	1.000
	(+)	4	1	
Diabetes	(-)	70	25	0.254
	(+)	32	18	
Corticosteroid	(-)	71	30	1.000
	(+)	31	13	
Type of antiresorptive agents	Bisphosphonate	55	15	*0.023
	Denosumab	30	23	
	Both	17	5	
Administration period (months)	Median/25-75% teil	22/16-25	19/9-36	*0.017
Separation of sequestrum	(-)	59	23	0.711
	(+)	41	19	
Osteolysis	(-)	6	13	*<0.001
	(+)	94	29	
Periosteal reaction	(-)	99	34	*<0.001
	(+)	2	8	
Osteosclerosis	(-)	26	15	0.126
	Uniform type	47	12	
	Mixed type	27	15	
Leukocytes (/μL)	Median/25-75% teil	6200/4500-8050	6000/4600-8700	0.347
Neutrocytes (/μL)	Median/25-75% teil	3883/2738-5634	4260/2817-6707	0.531
Lymphocytes (/μL)	Median/25-75% teil	1260/943-1738	1224/1040-2080	0.085
Neutrophil-lymphocyte ratio	Median/25-75% teil	2.84/2.01-5.40	2.73/2.00-5.14	0.491
Albumin (g/dL)	Median/25-75% teil	3.9/3.50-4.20	3.6/3.40-3.90	0.051
Creatinine (mg/dL)	Median/25-75% teil	0.76/0.64-1.01	0.82/0.72-1.22	*0.023

Multivariate analysis using the variable that was significant in the univariate analysis as a covariate showed that patients with non-odontogenic MRONJ had no osteolysis (p<0.001, odds ratio: 0.113, 95% confidence interval: 0.033-0.387) and periosteal reaction (p=0.005, odds ratio: 13.073, 95% confidence interval: 2.137-79.990) (Table 3).

**Table 3:** Differences of characteristics between odontogenic and non-odontogenic MRONJ: multivariate analysis Multiple logistic regression analysis was used. * means significant value MRONJ: medication-related osteonecrosis of the jaw

Variable		P-value	OR	95% CI
Osteolysis	(+)/(-)	*<0.001	0.096	0.030-0.312
Periosteal reaction	(+)/(-)	*0.004	11.983	2.169-66.203
Creatinine	mg/dL	0.078	1.754	0.938-3.276

## Discussion

This study showed that non-odontogenic MRONJ is devoid of osteolysis on CT imaging and shows periosteal reaction more frequently compared with odontogenic MRONJ.

Although the pathogenesis of MRONJ is not yet fully understood, the 2022 American Association of Oral and Maxillofacial Surgeons (AAOMS) Position Paper [4] stated that ARA and inflammation/infection are necessary for the development of MRONJ. It also stated that local risk factors include dentoalveolar operations, such as tooth extraction, and pre-existing inflammatory dental diseases, such as periodontal disease, periapical pathology, and mucosal irritation in denture wearers. Since many authors stated that dental infection is one of the primary risk factors for MRONJ manifestation, its complete removal before ARA initiation is considered significant. However, MRONJ often occurs in patients with no teeth, bony prominence, or denture use.

AAOMS 2022 [4] describes three hypotheses for the pathogenesis of MRONJ. 

The first is bone remodeling inhibition by ARA. ARA inhibits bone remodeling, causing bone cells to reach the end of their lifespan without being replaced by new bone, leading to necrosis and eventually MRONJ development. This mechanism is considered the central hypothesis of the pathophysiology of MRONJ. 

The second is local infection. In many animal studies, inflammation or infection of the jawbone causes MRONJ, similar to that observed in humans [8-15]. The presence of bacteria in necrotic bone in clinical specimens and the spread of necrotic bone with the severity of inflammation suggest that bacterial infection may be involved in the development and onset of MRONJ [14-17]. 

The third is angiogenesis inhibition, in which ARA directly inhibits bone remodeling and angiogenesis in several laboratory studies, suggesting that ARA may promote the onset of MRONJ by cutting off the supply of bone to the body [18-24]. The first and third hypotheses, that is, bone remodeling and angiogenesis inhibition are the causes of MRONJ, are similar to the hypothesis by Lesclous et al. [5]. They proposed that ARA causes aseptic osteomyelitis, which progresses to necrosis that reaches the oral mucosa, and later oral bacteria invade the exposed necrotic bone, and infectious symptoms appear. Dental infections are not essential in this pathogenesis. However, if the second mechanism, local infection, is the primary cause of MRONJ, there may be a prior dental infection such as periodontal disease, root canal lesions, or pressure ulcers due to incompatible dentures at the site where MRONJ develops. Therefore, in this retrospective study, we investigated whether dental infections preceded the onset of MRONJ. Consequently, 102 of the 145 MRONJ cases were thought to have developed in association with obvious dental infections. In the remaining 43 patients, there was no obvious local infection on radiographs, but 15 were denture wearers, two had bony prominences, and one had repetitive gingival occlusal irritation by the opposing teeth. Owing to the retrospective nature of the study, it was not possible to determine the degree of denture fit or pressure ulceration in denture users. The cause of the disease was unknown in 25 patients (17.2%) who developed the disease at a toothless site and did not use dentures; moreover, there were no obvious local factors, such as odontogenic infection, in these cases.

The results of a study on the differences in characteristics between odontogenic and non-odontogenic MRONJ showed that, in univariate analysis, non-odontogenic MRONJ was more common in patients treated with high-dose DMB. Multivariate analysis revealed that non-odontogenic MRONJ was significantly more common in patients with osteolysis or periosteal reactions on CT images. We recently found that high-dose DMB-treated patients often do not show osteolysis on CT imaging, which we termed non-osteolytic MRONJ [25,26].

Suyama et al. also reported a case of non-osteolytic MRONJ and found that it was difficult to determine the extent of bone resection at surgery from preoperative images and the prognosis tended to be poor [7]. BP is absorbed by osteoclasts as they resorb bone and leads to apoptosis, whereas DMB inhibits RANKL, which is necessary for osteoclast development, and prevents the formation of mature osteoclasts, thereby suppressing bone resorption. DMB may have a stronger inhibitory effect on bone metabolism; therefore, DMB-treated patients may be more prone to aseptic osteonecrosis, especially at high doses.

**Figure 2:**
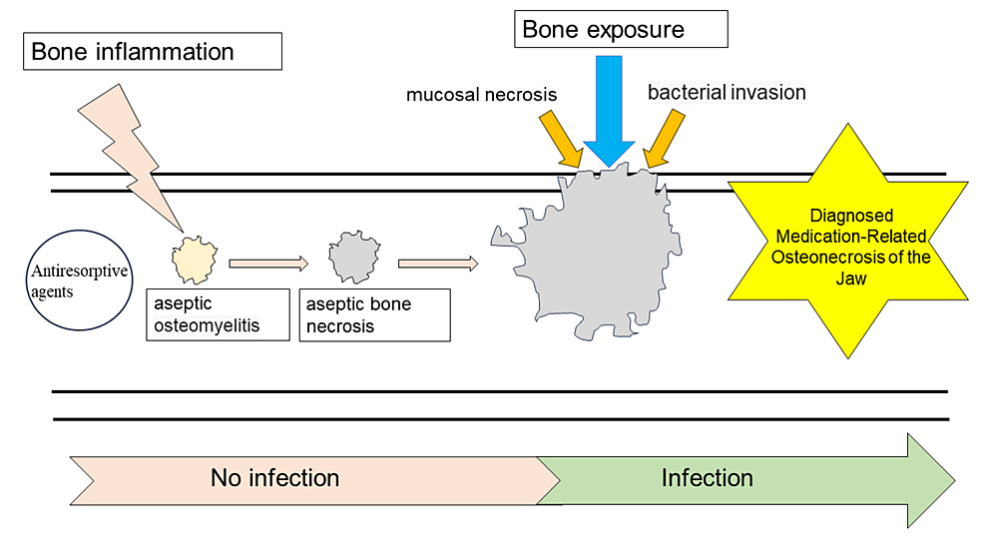
Hypothesis of MRONJ pathogenesis: preceding osteonecrosis, non-odontogenic type Image Credit: Yuki Sakamoto MRONJ: medication-related osteonecrosis of the jaw

We proposed the following three patterns of pathogenesis for MRONJ. The first is the preceding osteonecrosis, non-odontogenic type: aseptic osteonecrosis resulting from aseptic osteomyelitis caused by ARA expands, resulting in the loss of cortical bone and oral mucosa, and bone exposure. Finally, the exposed bone is infected with intraoral bacteria, resulting in MRONJ. This hypothesis is similar to the mechanism reported by Lesclous et al. [5], but aseptic osteonecrosis is rarely experienced in clinical practice, and this pattern of onset is considered rare (Figure 2).

**Figure 3:**
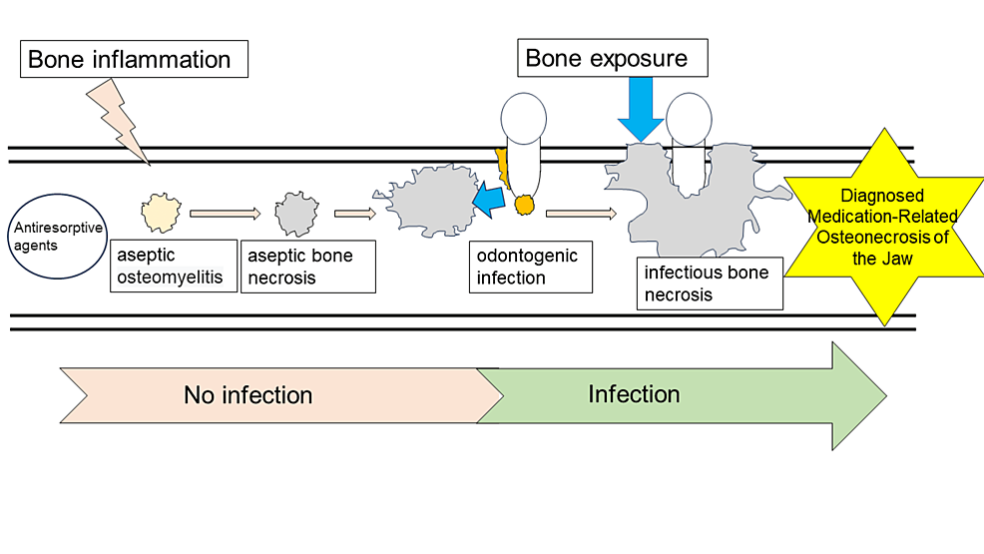
Hypothesis of MRONJ pathogenesis: preceding osteonecrosis, odontogenic type Image Credit: Yuki Sakamoto MRONJ: medication-related osteonecrosis of the jaw

The second is the preceding osteonecrosis, odontogenic type: similar to type 1, aseptic osteonecrosis resulting from aseptic osteomyelitis caused by ARA spreads to dental infection or infection from mucosal ulceration due to a bony ridge or ill-fitting dentures, resulting in MRONJ (Figure 3).

**Figure 4:**
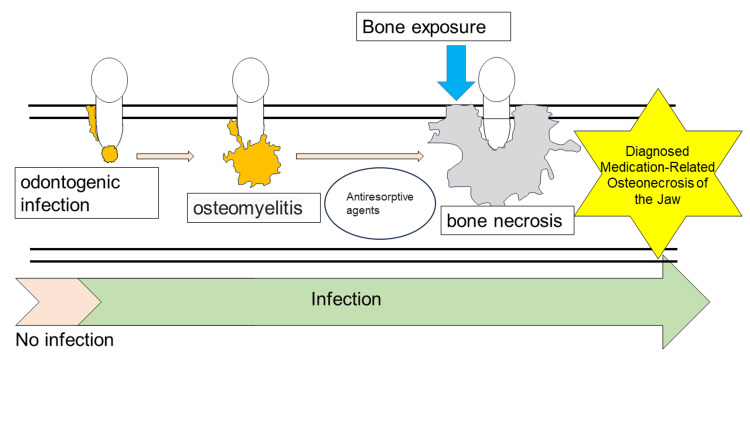
Hypothesis of MRONJ pathogenesis: preceding infection type Image Credit: Yuki Sakamoto MRONJ: medication-related osteonecrosis of the jaw

The third is the preceding infection type: bacterial osteomyelitis is caused by dental infection, followed by osteonecrosis due to the effect of ARA, which results in MRONJ (Figure 4).

Among these types, the preceding osteonecrosis, non-odontogenic type, was considered non-odontogenic MRONJ, in contrast to the preceding osteonecrosis, odontogenic type, and preceding infection types, which were considered odontogenic MRONJ in the present study. In cases of preceding osteonecrosis, non-odontogenic type, odontogenic infection is not related to the pathogenesis of MRONJ. This was more common in patients treated with high-dose DMB. This type of osteonecrosis occurs in areas without teeth, tori, or ill-fitting dentures. Preceding osteonecrosis, non-odontogenic type, is characterized by osteonecrosis that does not initially manifest as osteolysis because the onset of osteonecrosis is not caused by an infected alveolar lesion but by osteomyelitis in the body of the bone. However, as the lesion expands, it can easily reach the cortical bone of the body of the bone, which is likely to produce a periosteal reaction. Preceding osteonecrosis, odontogenic type, also appears to be more common in high-dose DMB-treated patients; however, it is often difficult to distinguish it from the preceding infection type because odontogenic infection is also a mechanism of pathogenesis. Regarding the prevention of MRONJ, it is important for the latter two types to eliminate dental infections prior to ARA administration and prevent the development of teeth as a source of infection through regular dental follow-up. However, in the case of preceding osteonecrosis, non-odontogenic type, it is difficult to prevent the development of MRONJ through dental checkups.

Limitations

This study has several limitations. This was an observational study with a small number of cases, and it is not clear whether the results obtained can be generalized. Owing to the retrospective nature of the study, the involvement of dental infection was based on radiographic images only, and the actual intraoral cavity was not examined carefully. Therefore, local infections that do not appear on the radiographic images may have been missed.

## Conclusions

Non-odontogenic MRONJ does not have an obvious preceding odontogenic infection. This type of MRONJ tends to occur more frequently in patients treated with high-dose DMB, and there were significantly more cases of non-osteolytic MRONJ radiographic evidence of no osteolysis or periosteal reactions. This study proposed three mechanisms for the pathogenesis of MRONJ: (1) preceding osteonecrosis, non-odontogenic type; (2) preceding osteonecrosis, odontogenic type; and (3) preceding infection type. However, the clinical significance of the proposed classification of pathogenesis is unknown. Therefore, we would like to study a large number of cases at several institutions and discuss the appropriate treatment and prognosis for each type of disease.
